# Overexpression of SAPCD2 correlates with proliferation and invasion of colorectal carcinoma cells

**DOI:** 10.1186/s12935-020-1121-6

**Published:** 2020-02-06

**Authors:** Yage Luo, Lili Wang, Wenwen Ran, Guangqi Li, Yujing Xiao, Xiaonan Wang, Han Zhao, Xiaoming Xing

**Affiliations:** 1 Department of Pathology, The Affiliated Hospital of Qingdao University, 16 Jiangsu Road, Qingdao, 266000 Shandong People’s Republic of China; 2 Department of Pathology, Qingdao University Basic Medicine College, Qingdao, 266000 Shandong People’s Republic of China

**Keywords:** Colorectal carcinoma, SAPCD2, Proliferation, Migration, Invasion, Cell cycle

## Abstract

**Background:**

Suppressor anaphase-promoting complex domain containing 2 (SAPCD2) is a novel gene playing important roles in the initiation, invasion, and metastasis of several malignancies. However, its role in colorectal carcinoma (CRC) still remains unclear.

**Method:**

In this study, we investigated the expression and biological function of SAPCD2 in CRC. Immunohistochemistry (IHC) for SAPCD2 was performed in 410 pairs of CRC specimens and corresponding normal epithelial tissues, and in 50 adenoma tissues. Clinical pathological factors were analyzed in relation to the expression of SAPCD2. The biological functions of SAPCD2 in CRC cells and its effect on cell cycle were investigated in vitro and in vivo through gain/loss-of-function approaches.

**Results:**

IHC showed that SAPCD2 expression was significantly higher in CRC tissues compared to adenoma and normal epithelium tissues and was correlated with tumor location (*p *= 0.018). SAPCD2 significantly promoted cell proliferation, migration, and invasion both in vitro and in vivo (*p *< 0.05). In addition, SAPCD2 knockdown in CRC cells was associated with reduced G_1_/S transition, while overexpression caused G_2_/M phase arrest (*p *< 0.05).

**Conclusions:**

In sum, SAPCD2 is overexpressed in CRC tissues and plays a critical role in CRC progression. Therefore, it might represent a promising therapeutic target for CRC treatment.

## Background

Colorectal cancer is one of the most common malignancies in the world, and its annual incidence in individuals younger than 55 years has continuously increased by almost 2% from the mid-1990s to 2014 [1]. However, the mechanisms regulating the development of colorectal cancer still remain elusive.

Suppressor anaphase-promoting complex domain containing 2 (SAPCD2), also known as p42.3 or C9orf140, is a cell cycle-dependent gene which was first identified in the gastric cancer (GC) cell line, BGC823 [2]. It is located in human chromosome 9q42.3 and encodes a 389-amino acid protein (42.3 kDa) with functional CC-domains at the C-terminus and an N-terminal EF-Hand domain [3–5].

Recently, several studies have reported that SAPCD2 is overexpressed in several kinds of solid tumors, including GC, hepatocellular carcinoma (HCC), melanoma, glioblastoma, and renal cell cancer (RCC) [2, 6–9], and might play important roles in cell proliferation, migration, and invasion by activating JAK/STAT, MAPK, and Wnt signaling pathways [3, 4, 7, 9]. Bioinformatics analysis in gastric cancer predicted a role of SAPCD2 in a signaling network also comprising S100A11, RAGE, P38, MAPK, microtubule-associated protein, spindle protein, and centromere protein, regulating cell proliferation, or in the Ras-Raf-1-MEK-MAPKK-MAPK pathway [3–5]. However, no functional experiments has confirmed this hypothesis. To date, little is known about the function of SAPCD2 in colorectal carcinoma (CRC).

In the present study, we conducted a retrospective research to investigate the relationship between the level of SAPCD2 expression and the clinical characteristics and overall survival (OS) of 410 Chinese Han CRC patients. In addition, we evaluated the biological role of SAPCD2 in cell proliferation, migration, invasion, and cell cycle in RKO and/or HCT116 cell lines. These findings provide new insights into the functions of SAPCD2 and its role in CRC.

## Materials and methods

### Tissues and cell lines

Four hundred and ten CRC specimens with matched adjacent normal epithelium tissues and 50 colorectal adenoma tissues were collected at the Affiliated Hospital of Qingdao University from 2014 to 2016. The tissues were obtained during colorectal surgery and were immediately fixed with 40 g/L formaldehyde and embedded in paraffin wax. Tissue microarray was constructed by TMAjrTM according to the manufacturer’s instructions (Pathology Devices, MD, USA). For quantitative real-time PCR (qRT-PCR) and western blot (WB) analysis, 20 sets of primary CRC tissues and distant normal epithelium tissues were obtained during colorectal cancer resection. Tissues were stored at − 80 °C for further use. None of the patients had received radiotherapy and chemotherapy before surgery. The protocol of this study and the informed consents were approved by the Ethics Committee of the Affiliated Hospital of Qingdao University.

Human CRC RKO and HCT116 cell lines (ATCC; Manassas, VA, USA) were cultured in Dulbecco’s modified Eagles’ medium (DMEM; Corning, 10-013-CVR, NY, USA) supplemented with 10% fetal bovine serum (FBS; Gibco, 16,000-044, NY, USA) and maintained at 37 °C in a humidified atmosphere with 5% CO_2_.

### Immunohistochemistry

Paraffin-embedded tissues were deparaffinized and rehydrated by xylene and a graded ethanol series. Sections were treated with 3% hydrogen peroxidase (10 min, room temperature), and antigen retrieval was performed with citrate buffer (pH = 6.0) for 35 min, followed by incubation with rabbit anti-SAPCD2 polyclonal antibody (1:1500; Abcam, ab126432, MA, USA) overnight at 4 °C. Then, sections were incubated with polymer-HRP secondary antibody (ZSGB-Bio, MA2522, Beijing, China) for 20 min at room temperature and developed by a 3,3-diaminobenzidine (DAB) kit (ZSGB-Bio, ZL1-9017, Beijing, China), counterstained with hematoxylin.

All slides were examined and graded by two pathologists blinded to the clinical diagnosis. Cytoplasmic SAPCD2 was quantified based on the extent of positive tumor cells and the intensity of staining. The percentage of positive tumor cells was scored as 0 (negative), 1 (1−25%), 2 (26–50%), 3 (51–75%), and 4 (> 75%). The staining intensity was scored as 0 (no staining), 1 (weak staining), 2 (moderate staining), and 3 (strong staining). The two scores were multiplied and the resulting immune-reactive score (IRS) (values from 0 to 12) was used to classify the samples into two categories: high level (4–12 scores) and low level (1–3 scores) [10].

### Quantitative real-time PCR analysis

Total RNA was extracted from RKO and HCT116 cell lines by using Trizol (Pufei, 3101-100, Shanghai, China), the mRNA of SAPCD2 was examined by using SYBR Master Mixture (TAKARA, DRR041B, Dalian, China) and signals were measured by a LightCycler^®^480 System (Roche, Basel, Switzerland). Total RNA of 20 pairs of fresh tissues was extracted by using the RNAprep Pure Tissue Kit (TIANGEN Biotech, DP140916, Beijing, China), according to the manufacturer’s instructions. Reverse transcription was performed using 1000 ng of total RNA using the PrimeScriptTMRT reagent Kit (TAKARA, RR047A, Dalian, China), the mRNA expression of SAPCD2 was examined by using SYBR^®^ Green PCR Master Mix (TAKARA, RP820A, Dalian, China) with gene-specific primers and real-time PCR Detection System (ABI, 7500, CA, USA).

For the analysis of SAPCD2 expression in RKO and HCT116 cell lines, the primer sequences were as follows: SAPCD2: 5′-GAGGTGACCGAGAAGAGTGAG-3′ (F) and 5′-GATGAAGGTGGAATCCAGAGG-3′ (R); GAPDH: 5′-TGACTTCAACAGCGACACCCA-3′ (F) and 5′-CACCCTGTTGCTGTAGCCAAA-3′ (R). The thermal cycling conditions were as follows: pre-denaturation at 95 °C for 30 s, 40 cycles of denaturation at 95 °C for 5 s, annealing at 60 °C for 30 s.

For the analysis of SAPCD2 expression in human tissues, the primer sequences were as follows: SAPCD2: 5′-GCTGAAGCAGATGAAGGAGCTGGAG-3′ (F) and 5′-ACCGGGCCACCTCTTGTACCT-3′ (R); GAPDH: 5′-CTGACTTCAACAGCGACACC-3′ (F) and 5′-TGCTGTAGCCAAATTCGTTGT-3′ (R). The thermal cycling conditions were as follows: pre-denaturation at 95 °C for 30 s, 40 cycles of denaturation at 95 °C for 10 s, annealing at 60 °C for 30 s, and extension at 72 °C for 1 min.

The SAPCD2 mRNA expression was measured by threshold cycle values (Ct). The results were calculated using the 2^−∆∆Ct^ method, and presented as fold changes.

### Western blot analysis

Cells were lysed in RIPA buffer (CW2333S) supplemented with protease inhibitor cocktail (CW2200S) and phosphatase inhibitors (CW2383S). Total protein was extracted from 20 paired tissue samples by Tissue Protein Extraction Kit (CW0891M) (all from CWBIO, Beijing, China) and quantified with the Ultrospec^®^ 3300 Pro. Total proteins from cells (30 μg) and tissues (10 μg) were separated by 10% SDS PAGE and transferred to a polyvinylidene fluoride (PVDF) membrane (Millipore, IPVH00010, MA, USA) at 300 mA for 110 min. After blocking with Tris-buffered saline containing Tween-20 (TBST, 1000:1) and 5% fat-free milk for 2 h, the membranes were incubated at 4 °C overnight with anti-SAPCD2 rabbit polyclonal antibody (1:1000; ab150707) and anti-GAPDH rabbit polyclonal antibody (1:4000; ab9485), and subsequently probed with secondary goat anti-rabbit antibodies (1:5000; ab6721) (all from Abcam, MA, USA) at room temperature for 1 h. Signals were analyzed by a chemiluminescence system (Thermo Fisher Scientific, MA, USA).

### Plasmid construction and transfection, and lentivirus transduction

The lentiviral vector containing the SAPCD2 sequence (GenBank accession number: NM_178448) was pLV-shSAPCD2, the control vector was pLV-shControl, and the SAPCD2 overexpression plasmid was lenti-SAPCD2. All vectors were purchased from Shanghai Gene Chem Corporation (Shanghai, China). The transfection of pLV-shSAPCD2 and pLV-shControl into RKO and HCT116 cells, and that of lenti-SAPCD2 overexpression and control plasmids into RKO cells were carried out according to the manufacturer’s instructions. The transfection efficiency was monitored by fluorescence microscopy (OLYMPUS, IX71, Tokyo, Japan) and stably transfected cells were selected by G418 at a concentration of 1 mg/ml.

### MTT assay

After transfection, RKO and HCT116 cells were seeded into 96-well plates at 1 × 10^3^/well and cultured for 1–5 days. MTT reagent (Genview, JT343, FL, USA) was added after 0, 24, 48, 72, 96, and 120 h and removed after a 4 h incubation at 37 °C. The formazan crystals were dissolved with dimethylsulfoxide (DMSO) (100 μl/well). The absorbance at 490 nm was measured using a microplate reader (Tecan Infinite, Mannedorf, Switzerland).

### Colony formation assay

For the colony formation assay, RKO and HCT 116 cells were seeded into 6-well plates at 1 × 10^3^ cells per well and cultured for 14 days. Colonies were fixed with ethanol and stained by GIEMSA for 20 min at room temperature.

### Celigo cell counting assay

After transfection, RKO and HCT116 cells were plated into 96-well plates at 1 × 10^3^/well and cultured for 1–5 days. Cells were counted by the Celigo automated cell counter (Nexcelom Bioscience, MA, USA), and cell growth curves were delineated.

### Cell cycle analysis

RKO and HCT116 cells were analyzed for DNA content by FACS Calibur flow cytometer (Millipore, MA, USA) according to previously described methods [11]. Briefly, cells were synchronized, harvested, and fixed with 70% ethanol at 4 °C overnight. After washing with ice-cold PBS, cells were stained with propidium iodide (PI, 50 μg/ml), mixed with Triton X-100 and RNase A for 30 min, and cell cycle was analyzed by a FACS Caliber flow cytometer (Beckman Coulter, CA, USA).

### Migration and invasion assays

A total of 1 × 10^5^ transfected cells were suspended in 200 μl serum-free medium and placed in the upper compartment of a transwell chamber (24-well; Corning, NY, USA). The lower chamber was filled with culture medium containing 15% FBS as a chemoattractant and incubated for 72 h. For the invasion assay, the inserts were pre-coated with extracellular matrix gel (BD Biosciences, MD, USA). After incubation, cells on the upper surface of the membrane were removed, and cells on the lower surface were fixed, stained with 0.1% crystal violet, and counted under a light microscope in five randomly picked fields.

### Animal experiments

A murine xenograft model was developed to investigate the effects of SAPCD2 on tumor invasion in vivo. Four-week old nude BALB/c-nu/nu mice were purchased from Shanghai Gene Chem Corporation. RKO cells were infected with pLV-shSAPCD2 or pLV-shControl, and 4 × 10^6^ infected cells were injected into the right flank. Tumor growth was examined from the third week for 10 days. Tumor volume (V) was monitored by measuring the length (L) and width (W) of the tumor following the equation: V = (L × W^2^) × 0.5. Protocols of animal experiments were approved by the Affiliated Hospital of Qingdao University.

### Statistical analysis

All data were evaluated using SPSS 19.0.0 software (SPSS, Chicago, IL, USA) and expressed as mean ± SD. All experiments were repeated at least 3 times. The relationship between the clinic pathological features and SAPCD2 expression, as well as the differences in SAPCD2 expression between normal epithelium, adenoma, and CRC tissues, were evaluated using a Chi square (χ2) test. Survival analysis was performed by the Kaplan–Meier method. Statistical significance was calculated by a Student’s two-sided t test and p values < 0.05 were considered as significant.

## Results

### SAPCD2 expression is elevated in CRC tissues

**Fig. 1:**
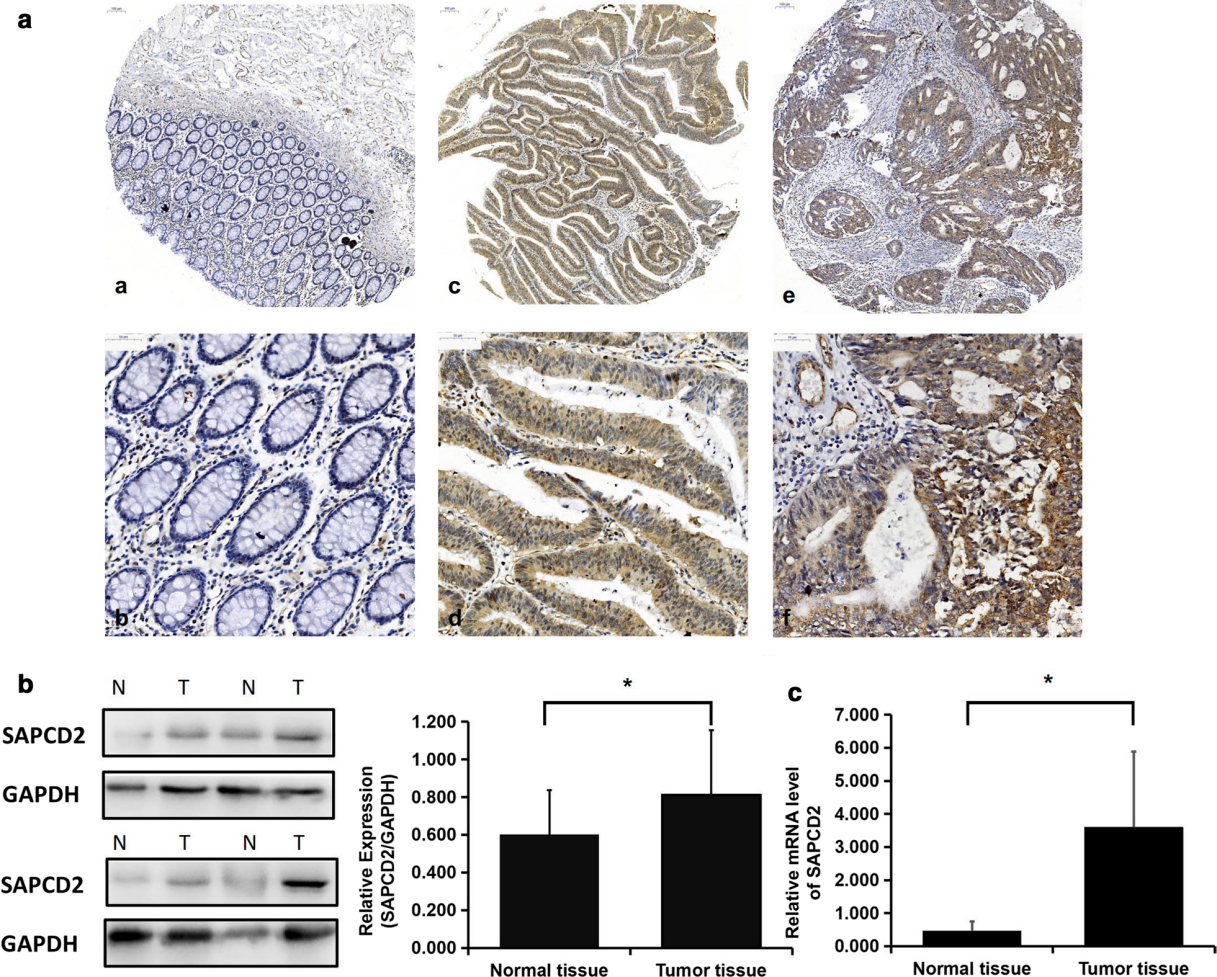
Expression of SAPCD2 in human tissues. **a** Immunohistochemical staining for SAPCD2 in normal epithelium, adenoma, and CRC tissues; **a**, **b** normal epithelium tissues; **c**, **d** adenoma tissues; **e**, **f** CRC tissues (**a**, **c**, **e**: magnification, × 100; **b**, **d**, **f**: magnification, × 400). **b**, **c** Detection of SAPCD2 in 20 pairs of CRC tissues and matched adjacent normal epithelium tissues. **b** WB analysis shows that the SAPCD2 protein was highly expressed in primary CRC tissues. **c** Quantitative real-time PCR analysis shows that SAPCD2 mRNA was more abundant in CRC tissues compared to normal epithelium tissues. (*N* normal epithelium tissues, *T* CRC tissues; *p < 0.05 compared to control)

IHC staining was performed to analyze the expression of SAPCD2 in CRC, adenoma, and normal epithelium tissues from 410 CRC and 50 adenoma patients. As shown in Fig. 1a, the expression level of SAPCD2 was significantly elevated in CRC tissues compared to normal epithelial tissues, and the protein mainly localized in the cytoplasm of CRC cells.

SAPCD2 protein and mRNA levels were further examined by WB and quantitative real-time PCR in 20 pairs of fresh CRC specimens and associated normal epithelium tissues. As shown in Fig. 1b, c, SAPCD2 protein and mRNA were significantly more abundant in CRC than in normal tissues (*p *= 0.045, *p *< 0.001, respectively). Moreover, the expression of SAPCD2 among the normal epithelium tissues, adenoma tissues and CRC tissues was obviously different (*p *< 0.001) (Table 1).

**Table 1:** SAPCD2 expression among the 50 adenoma tissues, 410 pair of CRC tissues and matched normal tissues

Tissue groups	n	SAPCD2 expression		*p* value
		High (%)	Low (%)	
Normal	410	29 (7.1)	381 (92.9)	
Adenoma	50	9 (18.0)	41 (82.0)	< 0.001
CRC	410	228 (55.4)	182 (44.6)	

### The expression of SAPCD2 is related to clinico-pathological characteristics but not with overall survival

To analyze the association between SAPCD2 expression and clinico-pathological features, we used a Chi square (χ2) test (Table 2). The expression level of SAPCD2 in the tumor samples of 410 patients was associated with left tumor location (p = 0.018), but not with gender, age, TNM stage, or lymph node metastasis.

**Table 2:** Correlations between the expression of SAPCD2 and clinic pathological characteristics (n = 410)

Characteristics	n	SAPCD2 expression		p value
		High (%)	Low (%)	
Gender				
Male	252	146 (57.9)	106 (42.1)	0.231
Female	158	82 (51.9)	76 (48.1)	
Age (years)				
≥50	364	204 (56.0)	160 (44.0)	0.619
<50	46	24 (52.2)	22 (47.8)	
Location				
Right colon	74	32 (43.2)	42 (56.8)	0.018
Left colon	336	196 (58.3)	140 (41.7)	
Tumor size (cm)				
≤ 6	342	184 (53.8)	158 (46.2)	0.098
> 6	68	44 (64.7)	24 (35.3)	
Tumor differentiation				
Well	18	11 (61.1)	7 (38.9)	0.070
Moderate	301	176 (58.5)	125 (41.5)	
Poor	91	41 (45.1)	50 (54.9)	
TNM stage				
I	55	28 (50.9)	27 (49.1)	0.078
II	138	77 (55.8)	61 (44.2)	
III	127	63 (49.6)	64 (50.4)	
IV	90	60 (66.7)	30 (33.3)	
Depth of invasion (T)				
T1	12	8 (66.7)	4 (33.3)	0.570
T2	67	36 (53.7)	31 (46.3)	
T3	149	88 (59.1)	61 (40.9)	
T4	182	96 (52.7)	86 (47.3)	
Lymph node metastasis (N)				
N0	229	127 (55.5)	102 (44.5)	0.945
N1 + N2	181	101 (55.8)	80 (44.2)	

**Fig. 2:**
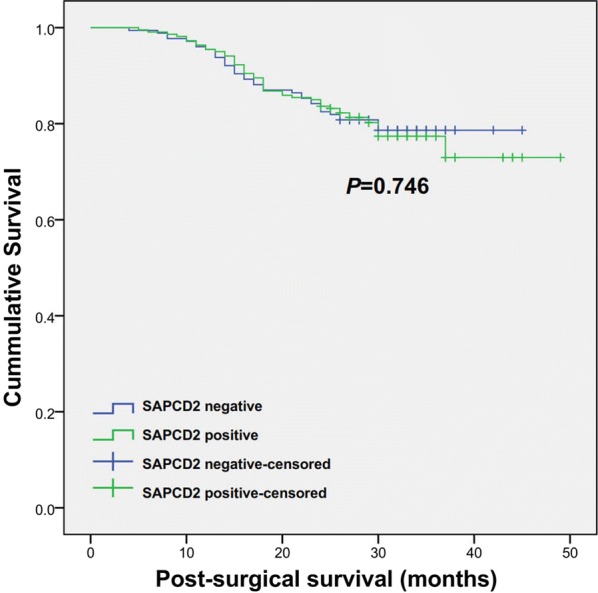
Overall survival (OS) curves of CRC patients. OS was correlated with the expression status of SAPCD2. No statistically significant difference was observed (*p *> 0.05)

Of the 410 CRC patients, 13 were lost to follow-up. The survival curves indicated that the expression of SAPCD2 was not related to OS (p = 0.746; Fig. 2).

### SAPCD2 promotes proliferation, migration, and invasion of CRC cells

**Fig. 3:**
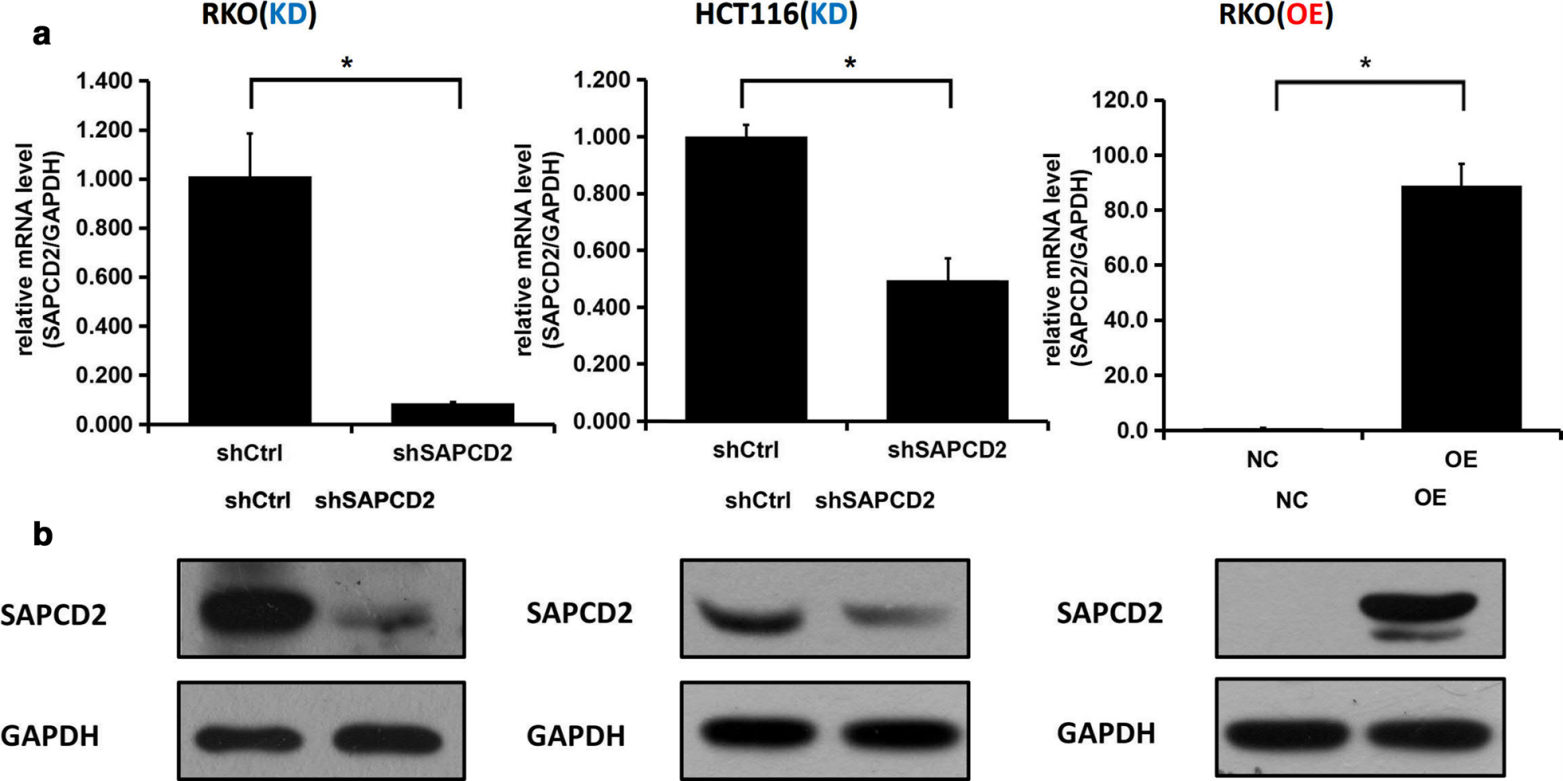
Expression of SAPCD2 in CRC cells. **a** Quantitative real-time PCR and **b** WB were performed in RKO and HCT116 cells after transfection with pLV-shSAPCD2, pLV-shControl, lenti-SAPCD2 overexpression, or control plasmid. (*KD* knockdown, *OE* overexpression; *p < 0.05 compared to control)

Both the pLV-shSAPCD2 silencing vector and the lenti-SAPCD2 overexpression vector were transfected into CRC cell lines. As shown by both quantitative real-time PCR and WB, SAPCD2 expression was significantly downregulated in pLV-shSAPCD2-transfected cells and upregulated in lenti-SAPCD2-transfected cells (all *p *< 0.05) (Fig. 3 a, b).

**Fig. 4:**
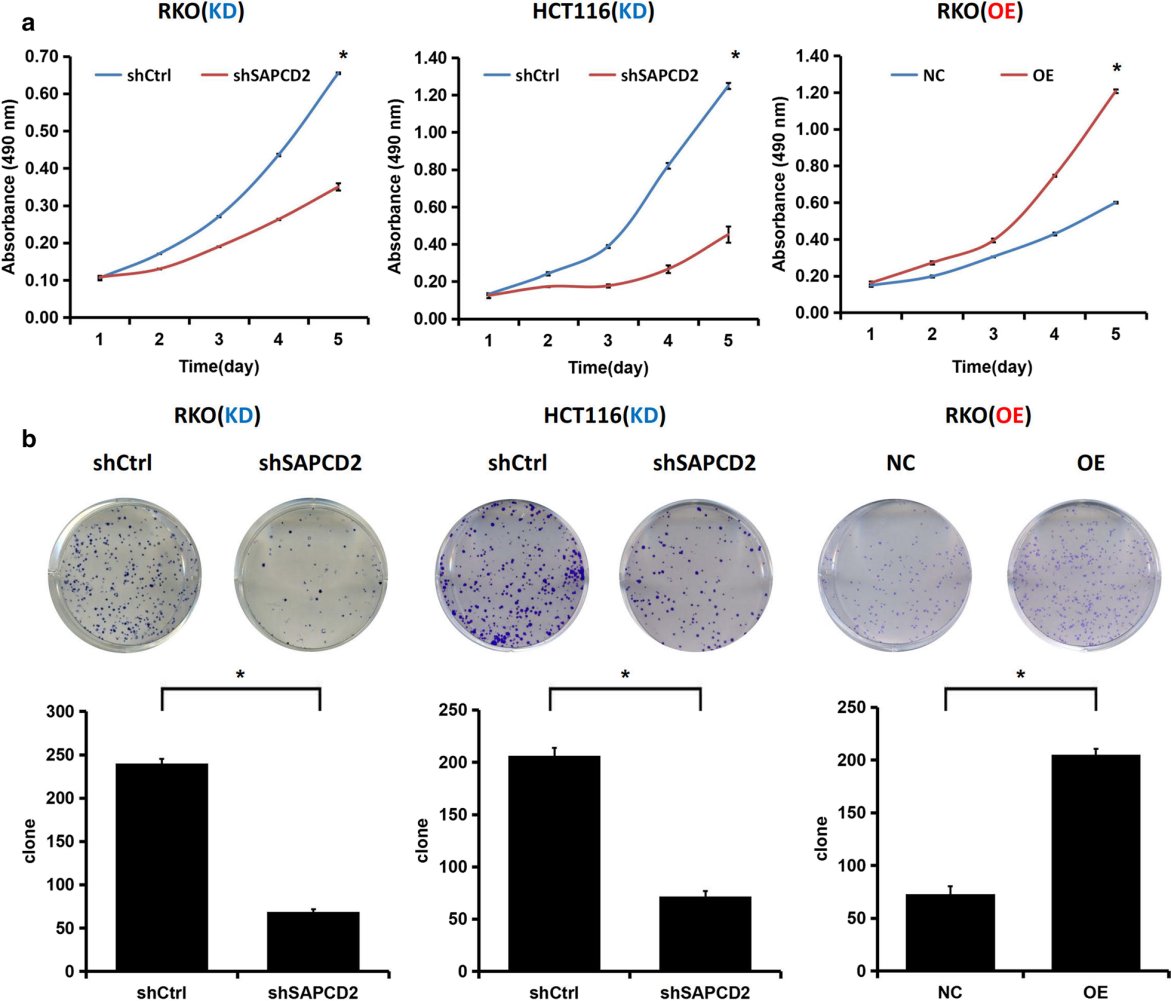

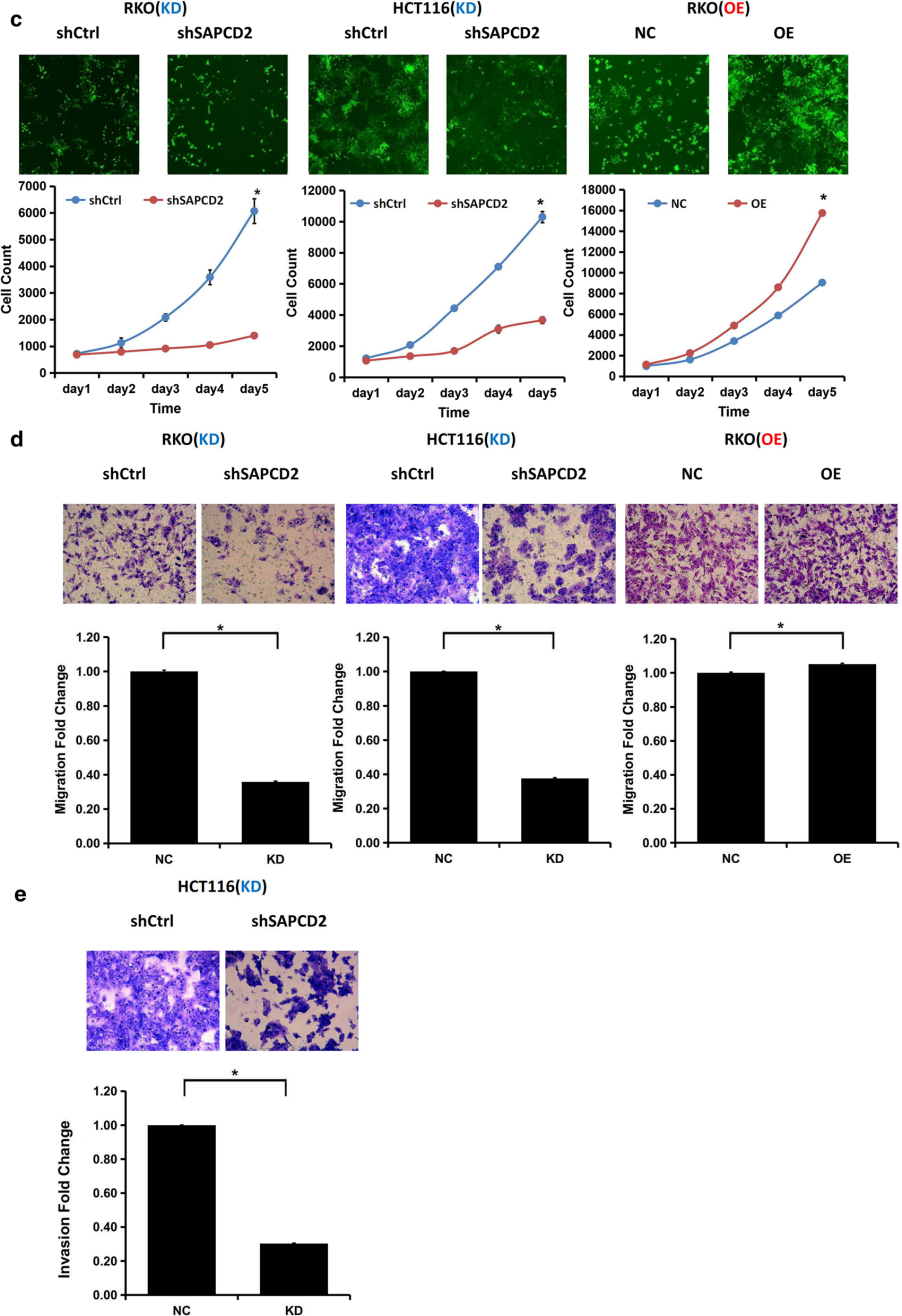
Effects of SAPCD2 on proliferation, migration, and invasion of CRC cells in vitro. Transfection of RKO and HCT116 cells with pLV-shSAPCD2 or pLV-shControl and transfection of RKO cells with lenti-SAPCD2 overexpression or the corresponding control plasmid. **a** MTT assay was performed at 5 days post-transfection. **b** Colony formation assay was conducted after transfection and further incubation for 14 days. **c** Celigo Cell counting assay was performed at 5 days post-transfection. **d** Transwell migration and **e** invasion were analyzed at 72 h post-transfection. All data are from three independent experiments and expressed as mean ± SD. (*KD* knockdown, *OE* overexpression; *p < 0.05 compared to control)

MTT and Celigo cell counting assays demonstrated that SAPCD2 knockdown significantly inhibited RKO and HCT116 cell proliferation, whereas SAPCD2 overexpression promoted cell proliferation in RKO cells (*p *< 0.05; Fig. 4a, c). The colony formation assay showed that SAPCD2 knockdown dramatically decreased the number of both RKO and HCT116 cell colonies, while its overexpression increased the colony number of RKO cells (*p *< 0.05; Fig. 4b). The cell migration assay showed an obvious decrease in RKO and HCT116 cell migration upon SAPCD2 knockdown, while SAPCD2 overexpression promoted RKO cell migration (*p *< 0.05; Fig. 4d). Moreover, SAPCD2 knockdown significantly reduced the invasion capability of HCT116 cells (*p *< 0.05; Fig. 4e). These results suggested that SAPCD2 was a crucial regulator of CRC cell proliferation, migration, and invasion.

### SAPCD2 induces the proliferation of CRC cells by affecting the cell cycle

**Fig. 5:**
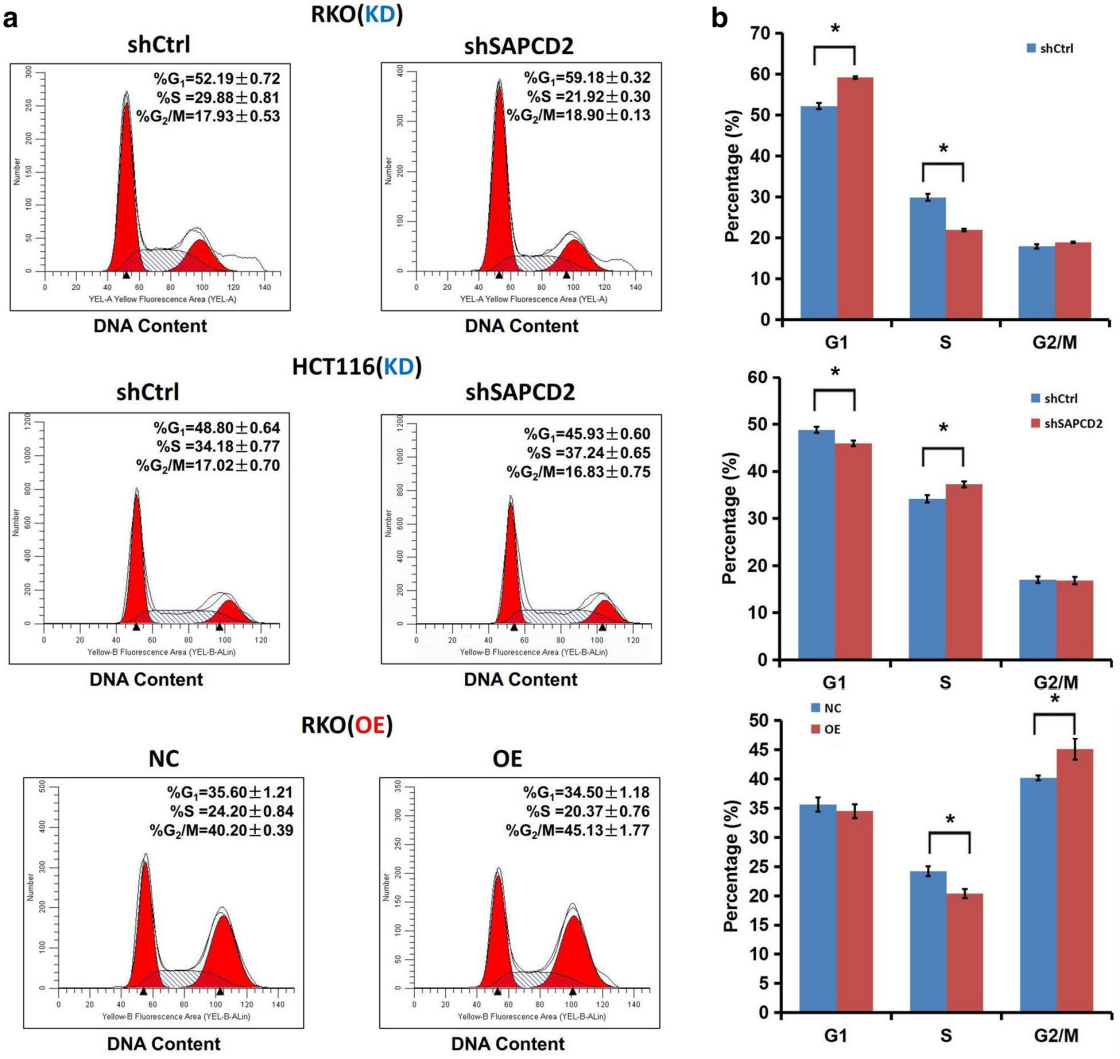
Impact of SAPCD2 on cell cycle. **a**, **b** Cell cycle profiles were analyzed after the transfection of RKO and HCT116 cells with pLV-shSAPCD2 or pLV-shControl, and transfection of RKO cells with lenti-SAPCD2 overexpression or control plasmid. All data are from three independent experiments and expressed as mean ± SD. (*KD* knockdown, *OE* overexpression; *p < 0.05 compared to control)

Flow cytometry was employed to investigate the effects of SAPCD2 on the cell cycle in RKO and HCT116 cells. We found that SAPCD2 knockdown lead to a significant peak of G_1_ phase and an obvious decrease in S phase in RKO cells, but a notably decrease in G_1_ phase and an increase in S phase in HCT116 cells. However, SAPCD2 overexpression led to an obvious decrease in S phase and an increase in G_2_/M phase in RKO cells (*p *< 0.05; Fig. 5). These results indicated that SAPCD2 was implicated in the inhibition of G_1_/S transition and in the promotion of G_2_/M phase arrest.

### SAPCD2 knockdown suppresses tumor growth in vivo

**Fig. 6:**
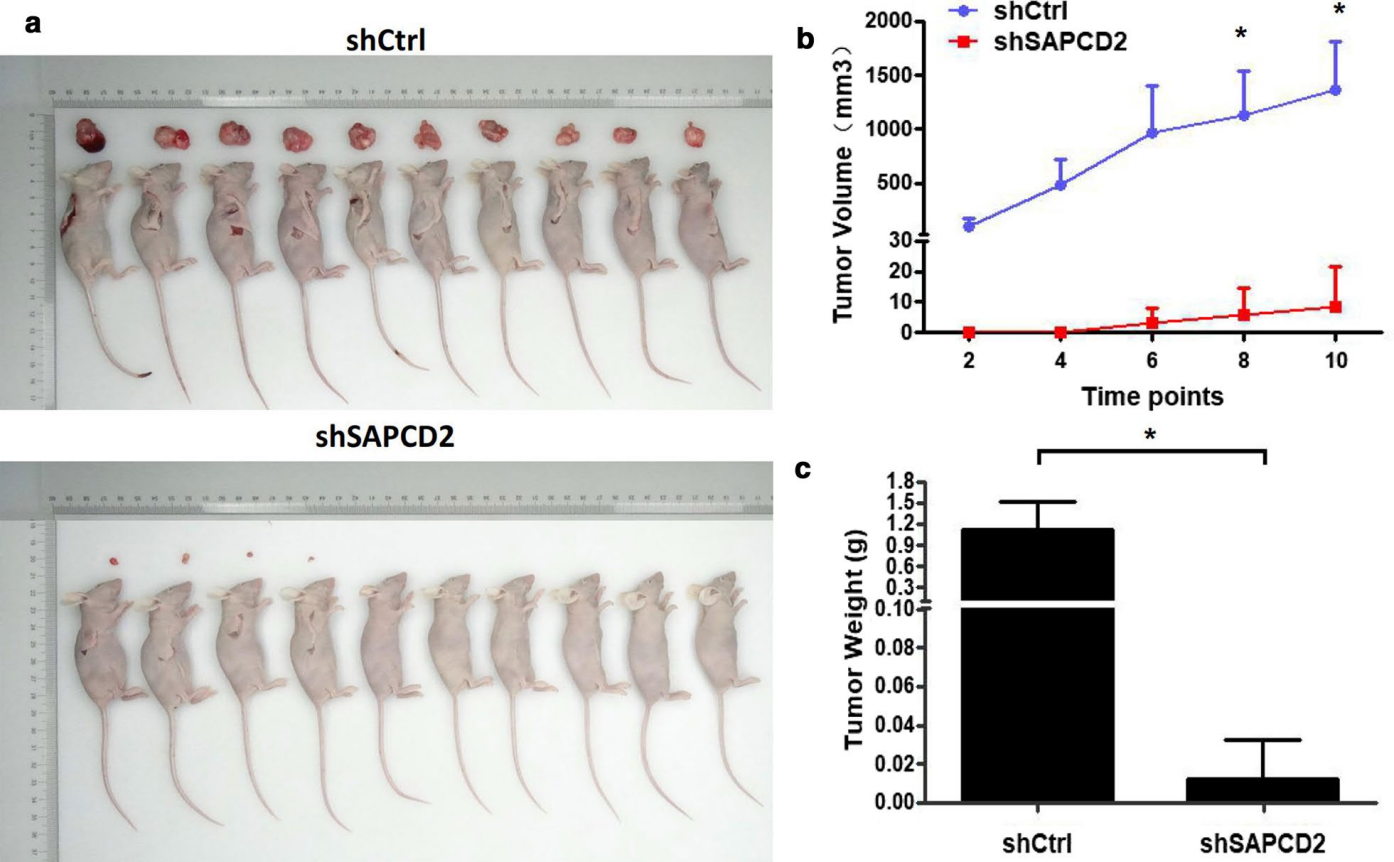
In vivo effects of SAPCD2 knockdown in a murine xenograft model. **a** Tumor formation in nude mice 24 days after injection with RKO cells transfected with pLV-ShSAPCD2 or pLV-shControl virus. **b** Volumes of the tumor generated in nude mice were measured every 2 days after the injection with RKO cells, starting from the third week. **c** Weight of tumors generated by transfected RKO cells 24 days after the initial injection. All data are from three independent experiments and expressed as mean ± SD. (*KD* knockdown, *OE* overexpression; n = 20; *p < 0.05 compared to control)

A murine xenograft model was developed by inoculating RKO cells, previously transfected with pLV-shSAPCD2 or pLV-shControl, into BALB/c nude mice, and tumor size was measured every 2 days from the third week. We found that tumors deriving from pLV-shSAPCD2-transfected cells showed smaller size (*p *< 0.001) and lower weight (*p *< 0.001) than did tumors generated by pLV-shControl-transfected cells (Fig. 6b, c). These results indicated that SAPCD2 knockdown suppressed tumor growth in vivo.

## Discussion

SAPCD2 has been reported to modulate malignant transformation and is considered as a potential biomarker of carcinogenesis [9, 10, 12, 13]. Consistently, it is upregulated in embryonic tissues, as well as in a number of human cancer cells, but not in normal tissues [14]. However, the biological functions of SAPCD2 in CRC are still unknown.

We found that SAPCD2 expression substantially differed in normal epithelium compared to adenoma and CRC tissues. Chen et al. reported that SAPCD2, besides being overexpressed in GC, was also associated with Helicobacter pylori inflammation and commonly expressed in chronic non-atrophic gastritis [15]. CRC usually develops from normal epithelium, which then transforms into adenoma and adenocarcinoma. Thus, adenoma is considered as a precancerous lesion [16, 17]. Therefore, our results indicated that SAPCD2 could be an oncogene implicated in early stages of the transition from normal epithelium to CRC. However, the exact function of SAPCD2 in this transition is unclear.

The expression of SAPCD2 has been reported to be related to gender, age, location, pathological classification, degree of infiltration, and the presence of lymphatic metastasis [10, 14]. Our study also showed that enhanced expression of SAPCD2 was significantly associated with left tumor location, as well as increased cell migration, invasion, and proliferation. A recent research reported that SAPCD2-negative CRC patients showed better survival [14]. However, in our study, no significant association between SAPCD2 expression and OS was observed. Further investigations, based on extended follow-up periods, need to be conducted to clarify this issue.

We found that SAPCD2 knockdown in RKO cells strongly inhibited cell proliferation and migration, SAPCD2 knockdown in HCT116 cells obviously inhibited cell proliferation, migration and invasion in vitro. Consistently, SAPCD2 silencing in RKO cells significantly reduced their in vivo tumorigenicity in nude mice. Conversely, SAPCD2 overexpression in RKO cells stimulated cell proliferation and migration. These results indicate a role of SAPCD2 in CRC progression.

Our findings support the hypothesis that SAPCD2 is involved in cell cycle regulation. SAPCD2 was initially identified by mRNA differential display (mRNADD) combined with cell cycle synchronization [2, 18]. Several studies have shown that the expression of SAPCD2 in G_1_ and M phases is higher than that during S and G_2_ phases [2, 3, 19], and cell cycle dysregulation is known to be associated with cancer progression [20, 21]. Previous studies have shown that SAPCD2 expression is closely associated with Cyclin B1 and Chk2, as SAPCD2 knockdown promotes the down-regulation of Cyclin B1 and up-regulation of Chk2, while SAPCD2 overexpression promotes the up-regulation of Cyclin B1 [6, 18]. As key cell cycle-dependent genes, Cyclin B1 and Cdc2 are involved in the G_2_-M phase transition, controlling the entry in M phase, exit, and promoting uncontrolled cell proliferation [22]. Chk2 is another key gene involved in M phase regulation, which phosphorylates and sequesters Cdc25 in the cytoplasm, thereby suppressing the dephosphorylation of CycliB1/Cdc2 and inhibiting mitosis [23, 24]. However, there were no changes in the expression of Cdc25 and Cdc2 [6, 19]. In this study, we observed that SAPCD2 knockdown was associated with the inhibition of the G_1_/S transition, while SAPCD2 overexpression led to arrest in G_2_/M phase. Does SAPCD2 overexpression cause G_2_/M phase arrest by upregulating Cyclin B1 and downregulating Chk2, which further result in the wrong chromosome segregation and mitotic progression? However, further investigation still need to ascertain the molecular mechanisms involved in the control of cell proliferation in G_1_ and G_2_/M phase.

The signaling pathways involved in SAPCD2-mediated regulation of solid tumor progression are still unknown. Epithelial–mesenchymal transition (EMT) is critical for tumorigenesis and is required for invasion and metastasis of various types of tumors [25–27]. Several studies have reported that SAPCD2 suppresses E-cadherin expression [9, 10, 12], and a reduced E-cadherin level is closely correlated with cancer progression and invasion. Previous bioinformatics analysis indicated that the expression of SAPCD2 in GC might be regulated by the MAPK pathway via the APC and members of the S100 family, which are highly homologous to the CC and EF-hand structural domain of SAPCD2 [3–5]. Notably, both S100 and APC are implicated in tumor progression [28–31]. A role of SAPCD2 in the promotion of tumor metastasis and EMT progression through Wnt, MAPK, and PI3K/AKT has been proposed in melanoma and renal cell cancer [7, 9]. Weng et al. have found that the inhibition of the JAK/STAT, MAPK, and Wnt signaling pathways downregulates the expression of SAPCD2, which was also found to be controlled by STAT5, EZH2, and β-catenin. Moreover, STAT5 enhanced the expression of SAPCD2 by recruiting EZH2 and β-catenin [10].

## Conclusion

In sum, our findings demonstrated that the expression of SAPCD2 is higher in CRC tissues than in normal epithelium and is involved in the “normal epithelium-adenoma–CRC” transition. SAPCD2 strongly affected cell proliferation, migration, and invasion. The effects of SAPCD2 on cell proliferation were correlated with G_1_/S inhibition and cell arrest in G_2_/M. Our study provides new evidence for the role of SAPCD2 in CRC. The elucidation of the precise molecular events responsible for the SAPCD2-mediated effects is imperative for the development of new therapies in CRC. Further studies should be conducted on the related signaling pathway with SAPCD2 in the progression of colorectal cancer.

## Data Availability

The datasets used and/or analyzed during the current study are available from the corresponding author on reasonable request.

## Abbreviations

SAPCD2: Suppressor anaphase-promoting complex domain containing 2
CRC: Colorectal carcinoma
GC: Gastric cancer
RCC: Renal cell cancer
HCC: Hepatocellular carcinoma
OS: Overall survival
IHC: Immunohistochemistry
qRT-PCR: Quantitative real-time PCR
WB: Western blot
PVDF: Polyvinylidene fluoride
SDS-PAGE: Sodium dodecyl sulfatepolyacrylamide gel electrophoresis
TBST: Tris-buffered saline tween-20
GAPDH: Glyceraldehyde-3-phosphate dehydrogenase
EZH2: Enhancer of zeste homolog 2
STAT5: Signal transducer and activator of transcription 5
EMT: Epithelial–mesenchymal transition
mRNADD: mRNA differential display
